# Supplementary material for: Interaction between a Novel Oligopeptide Fragment of the Human Neurotrophin Receptor TrkB Ectodomain D5 and the C-Terminal Fragment of Tetanus Neurotoxin
Source: Molecules. 2021 Jun 30;26(13):3988. doi: 10.3390/molecules26133988 (PMC8272241; doi:10.3390/molecules26133988)
Supplement: Supplementary file 1 [file molecules-26-03988-s001.zip › molecules-1202148-supplementary.pdf]

SUPPLEMENTAL MATERIAL (SM)

Interference between a novel Oligopeptide Fragment of the Human Neurotrophin Receptor TrkB ectodomain D5 and the C-terminal fragment of Tetanus neurotoxin

Ana Candalija, Thomas Scior , Hans-Richard Rackwitz, Jordan E. Ruiz-Castelan, Ygnacio Martinez-Laguna, José Aguilera

Triple mutant Hc-Mut with mutated residues Y266A K311A E343A

Affectation of the in vitro binding to the D5 peptide by the mutations suggests that HC-Mut may also have a minor ability to bind neuronal membranes and to be internalized. CGNs were incubated with fluorescent Hc-TeNT or Hc-Mut (500 nM each one) during 30 min at 4ºC for binding or during 2h at 37ºC for internalization. Cells were analyzed by flow cytometry and resulting histograms indicates that cells contain less quantity of Hc-Mut in comparison with Hc-TeNT, bound to the membrane or endocyted in the soma. In binding conditions there were approximately 10% fewer cells labeled with HcMut than labeled with Hc-TeNT. Similarly, in internalization assays cells show about 15% less labeling with HC-Mut than with wild type Hc-TeNT (Figure 5b). Binding and internalization assays with Hc-TeNT or with HC-Mut were also analyzed by confocal microscopy corroborating that the mutations in Hc-TeNT affects its capacity to bind and to be endocyted in CGNs, although the interaction was not completely abolished. Finally, we also proposed to determine whether the reduced binding and internalization of HC-Mut was correlated with a lower proportion of this fragment colocalizing with TrkB in neurons. CGNs were/was incubated with 10 nM of HC-A555 or 10 nM Hc-Mut during 30 min at 4 ºC for binding assays, and during 2h at 37ºC for uptake assays. Immunofluorescence was performed to detect TrkB. Representative confocal images for each condition were taken. Colocalizing analyzed pixels for Hc-TeNT and TrkB and colocalization

percentages were calculated. Results indicate that membrane-bound HC-Mut colocalized with TrkB about 60% less than did Hc-TeNT. In contrast, no significant reduction was found of colocalization in internalization conditions, thus indicating that although there is a lower quantity of HC-Mut inside the neurons, endocyted HC-Mut colocalizes with TrkB in a similar proportion than as does Hc-TeNT. The results are displayed (**Figure S1**).

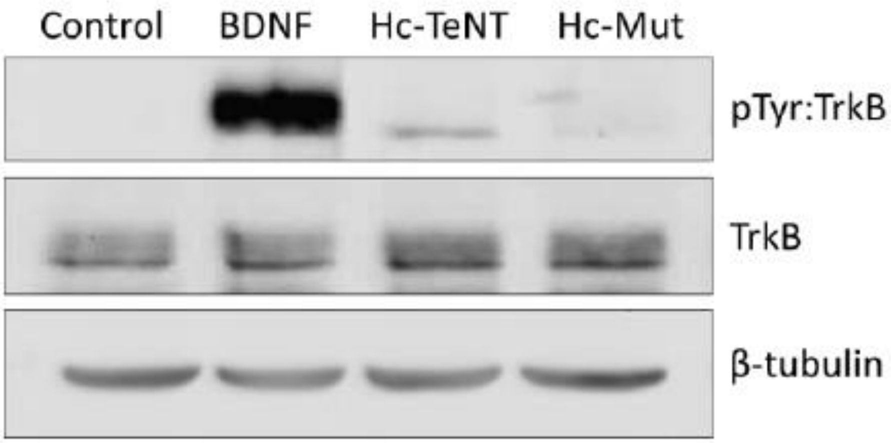

Figure S1. Levels of phosphorylated TrkB in CGN after treatment with Hc-TeNT or Hc-Mut. The cells were held serum-free prior to treatment with 100 nM Hc-TeNT, 100 nM HC-Mut, or 50 ng / mL BDNF. Immunoprecipitation with an antibody against phosphorylated tyrosinases, which are present in cell lysates, was performed and analyzed by Western Blot using an antibody against TrkB. Cell lysates were also analyzed for total TrkB and beta-tubulin levels.

The identification of a novel binding component

As working hypothesis, we assumed Trk to be that long-awaited third component for CNT recognition and internalization. Initially, two different CNT binding modes to Trk appeared plausible: (i) the NTs and CNTs would constitute molecular competitors at the D5 binding site (BS) for NTs; (ii) the CNTs would allosterically bind to either a Trk site other than the NT-BS on d5, or on a hitherto structurally unknown ectodomain other than D5 (**Figure S2**). At the time of modeling the entire protein surface was manually and computationally screened for potential PPI. Plausible solutions of the structural analysis [1], Escher NT [2] and Chimera [3]) were refined using a rotamer library for local or global settings (Chimera [3], Scwrl-4 [4]). In particular, the models were inspected for (i) shape complementarities, (ii) nonbonded binding complementarities, (iii) noncovalent bond strengths (salt bridges, semi-ionic or polar hydrogen bond networks versus single hydrogen bonds), (iv) topological features for PPI hot spots (ionic, polar and nonpolar patches), (v) loop or turn preferences for functional sites, (vi) correlation (or neighborhood) effects in close range. All six features could be brought on an equal footing for PPI modeling (**Figure S3**).

Prior to computation a workflow was elaborated listing the required available input data at the time of modeling. The input data influences the type of results to be expected as well as assumptions, applicability, or limitations to take into account at the time of molecular modeling (**Table S1**).

Binding model of the tetanus – neurotrophin receptor (NTR)

The protein - protein interface (PPI) between NTs (NGF, NT-3, NT-4/5) and NTRs (TrkA, B or C) was studied to draw conclusions about the postulated PPI between CNTs and Trk (cf. PDB data bank [5] entries: 1HCF [6], 1WWA [7], 1WWB [7], 1WWC [7] and 1WWW [8]). The intensity of research in the field of clostridial neurotoxins is reflected in the plethora of crystallographic information which entered the protein data bank PDB [5]. Concerning the ligands all crystal structures of Hc-TeNT were inspected (e.g. PDB codes: 1FV2 [9]; 1AF9 [10]; or 1A8D [11] = [1A80]) along with the complete structure of BoNT serotype B (PDB code: 1EPW [12]. The latter was used for homology modeling of the complete TeNT target, structurally unknown in its HCN and light chain domains because striking functional an structural similarities between TeNT and BoNT can be found in the literature [13]. The

crystal structure with PDB code 1HCF served as reference, i.e. template structure [6]. The contact zone comprises residues E1127, Y1129, K1174, S1201, Y1202, N1203, N1204, E1206, E1310, D1315 (**Figure S3**). Of note, the residue labels follow that of reference 1HCF. In addition, **Table S2** summarizes aspects for the proof of concept and the Figures S5 to S8 illustrate the findings concerning the proposed PPI (hotspot). By a BLAST search the homology of the Trk family for the same species or across the species barrier were studied [14–16]. Albeit, no species dependency for the short residue segment GCLQLDNPTHMNNG was necessary since that segment does not change for Mammalian animals and chicken birds (**Figure S4**).

<u>Synthesis of the cyclic peptide and negative control sequences</u>

The linear sequence was obtained during synthesis with its free amino- and carboxy-terminal groups (NH2-GCLQLDNPTHMNNGMLQLDNPTHCNNG-COOH). For controls a so-called "scrambled" sequence was taken (pep-d5-scr): NH2-GCNGHNDPLTHLMNLQ MDQTPNLCNNG-COOH. Both linear peptides as identified by mass spectrometry were oxidized to the corresponding cyclic disulfides (S-S bridge between Cys^{2} and Cys^{24}) by DMSO mediated oxidation.

In all cases, the linear peptides were dissolved in 5% acetic acid at a concentration of 1 mg/ml and the pH was adjusted to 6 with (NH4)2CO3. DMSO was added to a final concentration of 10% and oxidation was allowed to proceed for 24 hours at room temperature. The final reaction mixture was diluted twofold with 5% acetonitrile in 0.05% aq. TFA. The product was purified on a reversed phase C18 column as described above. Fractions were monitored by MALDI mass spectrometry and those containing the disulfide cyclized peptides were pooled and lyophilized.

Two more peptides showed similar properties in length and side chain chemistry and could serve as negative controls: NH2-SAPATGGVKKPHRYRPG-CO-amid or NH2-TGRGKGGKGLG KGGAKRHRKVLRD-COOH [17]. This segment is related to both ligands the carboxyl-terminal half (Hc-TeNT) [18] or the endogenous ligands NTs which play a physiological role [19]. A schematic view of ligand(s) and receptor complex formations with biological effects is presented (**Figure S2**).

Ligand(s) + Receptor	complex formations	biological effects
NT + Trk	==> NT-D5^{Trk} (crystal*)	==> cell uptake essential for life (observed)
NT + CNT + Trk	==> NT-D5^{Trk} + *CNT-Trk*	==> cell uptake, leading to death (observed)
NT + CNT + Trk + *AD*	==> NT-D5^{Trk} + *CNT-AD*	==> *no CNT but NT uptake, no death* (designed)

Figure S2. Antidote mechanism: Scheme of the biochemical reactions in absence or presence of clostridial bacteria neurotoxins (CNT) and novel antidote (AD). First line: the normal (physiological) events; second line: clostridial infection leading to death; third line: addition of the proposed antidote (AD) to prevent neurotoxin (CNT) internalization while neurotrophin ligands (NT) can still bind to their receptors (Trk). *Crystal structures of the NT binding site on the ectodomain D5 of Trk are known*. Words appear in Italics to symbolize the hypothetical character (H_O), i.e. not yet observed events as part of the present study.

Whenever possible, modeling was based on structural data, mostly the PDB entries 1HCF [6] and 1A8D [11] (**Figures S3** and **S4)**.

```
MKNLDCWVDNEEDIDVILKKSTILNLDINNDIISDISGFNSSVITYPDAQLVPGINGKAI 60
HLVNNESSEVIVHKAMDIEYNDMFNNFTVSFWLRVPKVSASHLEQYGTNEYSIISSMKKH 120
SLSIGSGWSVSLKGNNLIWTLKDSAGEVRQITFRDLPDKFNAYLANKWVFITITNDRLSS 180
ANLYINGVLMGSAEITGLGAIREDNNITLKLDRCNNNNQYVSIDKFRIFCKALNPKEIEK 240
                   1127E Y1129
LYTSYLSITFLRDFWGNPLRYDTEYYLIPVASSSKDVQLKNITDYMYLTNAPSYTNGKLN 300
*******************************************************
          K1174                   1201S 1204N E1206
IYYRRLYNGLKFIIKRYTPNNEIDSFVKSGDFIKLYVSYNNNEHIVGYPKDGNAFNNLDR 360
************************************************************
  R1226
ILRVGYNAPGIPLYKKMEAVKLRDLKTYSVQLKLYDDKNASLGLVGTHNGQIGNDPNRDI 420
************************************************************
 1289W   H1293        1310E    D1315
LIASNWYFNHLKDKILGCDWYFVPTDEGWTND                        1315=452
********************************
```

Figure S3. Display of computed PPI model with the proposed NTR-binding residues of TeNT. The positional numbers are given for the HCC fragment and the entire amino acid sequence of TeNT [1A8D] [11]; ******* = proposed 3D-model of the TeNT interface with NTR; underlined bold = proposed binding residues; underlined = reported key residues for the known lactose-binding and sialic acid-binding site.

```
        279 283             H299                        SK328
1WWA_X| -VQVNVSFPASVQLHTAVEMHHWCIPFSVDGQPAPSLRWLFNGSVLNETS
1WWC_A| VALTVYYPPRVVSLEEPELRLEHCIEFVVRGNPPPTLHWLHNGQPLRESK
1WWB_X| ----VHFAPTITFLESPTSDHHWCIPFTVKGNPKPALQWFYNGAILNESK
1HCF_X| ----SHMAPTITFLESPTSDHHWCIPFTVKGNPKPALQWFYNGAILNESK
                *  . *. .    . ** * * *:* *:*:*:.**  *.*:.
rat_d5| ----VHFAPTITFLESPTSDHHWCIPFTVRGNPKPALQWFYNGAILNESK
dog_d5| ----VHFAPTITFLESPTSDHHWCIPFTVKGNPKPALQWFYNGAILNESK
cow_d5| ----VHFAPTITFLESPTSDHHWCIPFTVKGNPKPALQWFYNGAILNESK
chicd5| ----VFFAPNITFIESPTPDHHWCIPFTVKGNPKPTLQWFYEGAILNESE
                *  . :. .    . ** * * *:* *:*:*:.:*  *.*:.

        Y329  H335   342 343 |DN350                      376
1WWA_X| FIFTEFLEPAANETVRHGCLRLNQPTHVNNGNYTLLAANPFGQASASIMA
1WWC_A| -I--IHVEYYQEGEISEGCLLFNKPTHYNNGNYTLIAKNPLGTANQTING
1WWB_X| YICTKIHV--TNHTEYHGCLQLDNPTHMNNGDYTLIAKNEYGKDEKQISA
1HCF_X| YICTKIHV--TNHTEYHGCLQLDNPTHMNNGDYTLIAKNEYGKDEKQISA
         *  .:      :    .*** :::*** ***:***:* *  *  .  * .
rat_d5| YICTKIHV--TNHTEYHGCLQLDNPTHMNNGDYTLMAKNEYGKDERQISA
dog_d5| YICTKIHV--TNHTEYHGCLQLDNPTHMNNGDYKLVAKNEYGKDEKQISA
cow_d5| YICTKIHV--TNHTEYHGCLQLDNPTHMNNGDYKLVAKNEYGKDEKQISA
chicd5| YICTKIHV--INQSEYHGCLQLDNPTHLNNGAYTLLAKNEYGEDEKRVDA
         *  .:      :    .*** :::*** *** *.*:* *  *  .  : .

        377   383           396
1WWA_X| AFMDNPFEFN-----------
1WWC_A| HFLKEPFPVDEVSPTPPITVT
1WWB_X| HFMGWPGID------------
1HCF_X| HFMGWPG--------------
        *:  *
rat_d5| HFMGRPGVDYETNP-------
dog_d5| HFMGWPGIDDG----------
cow_d5| HFMGWPGIDDG----------
Chicd5| HFMSVPGDGTGSGP-------
         *:  *
```

Figure S4. MSA of the Trk domain d5. Line 1: positional numbers of the residues in the liganded binding patch. Lines 2, 3, 4 and 5: crystal structures of human TrkA, C and B (two independent PDB entries). Line 6 and 11: symbols for sequence homology: (* : . for identity/higher/lower similarity); underlined bold = binding residue; bold face = homologous. Lines 7 to 10, TrkB domain D5 for common vertebrate species.

Superposition of the two analogous ligand proteins was carried out with ectodomain D5 of TrkB. At the time of modeling only the ligand-binding part D5 was elucidated structurally by crystallography. The PPI consists of three segments two of which contain Hydrogen bonds and are contoured (**Figures S5** , **S6** and **S7**).

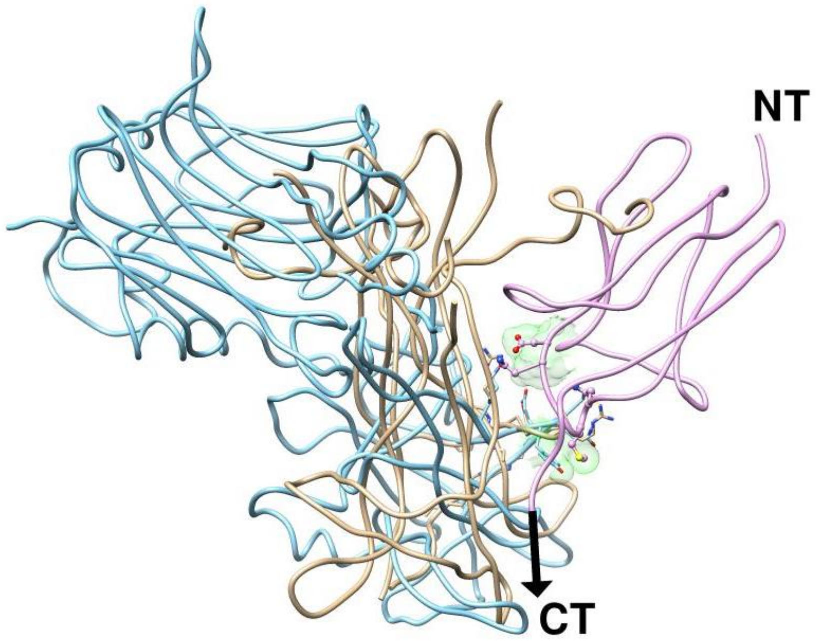

Figure S5. Detailed view of observed and predicted PPIs in superposition. The crystal complex between NT ligand (beige) and NTR (d5 of TrkB) (magenta) [1HCF] [6] was superposed on the proposed complex between Hc-TeNT (blue) and D5 (magenta). A hydrophobic zone forms part of the binding patch and is displayed by its contouring atomic surfaces (green).

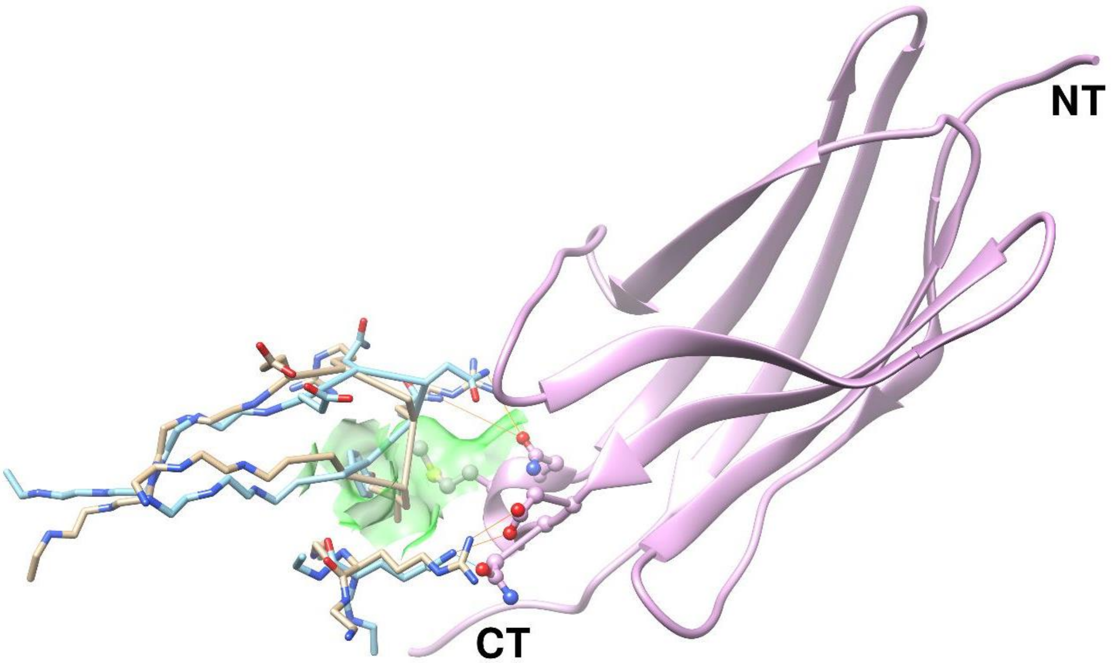

Figure S6. Orthogonal view of the binding patch between computed (blue) and observed (beige) predicted complexes. Following the beige (NT) and blue (TeNT) main chains, it can be judged by eyesight that the main chains of both proteins coincide to bring the binding residues in close proximity. They have analogous binding patches. They are neither identical nor homologous. The hydrophobic zone which forms part of the binding patch is displayed by the contours of participating atoms (green).

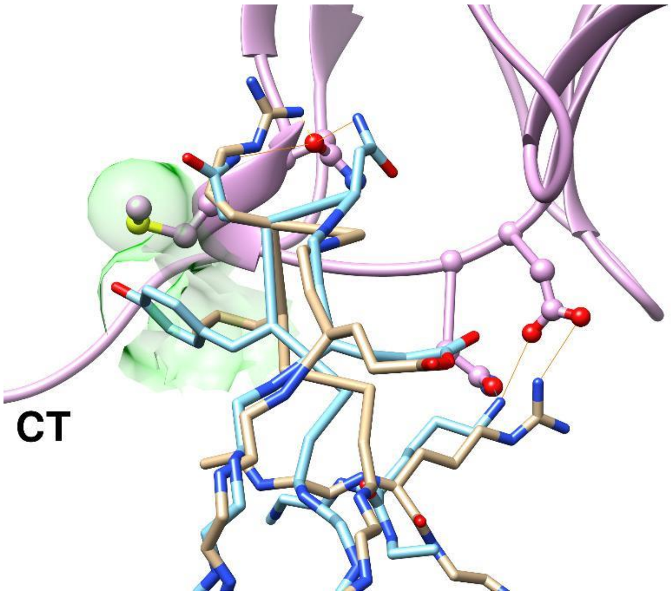

Figure S7. Annotated view of the superposed complexes. The interacting amino acids are labeled in one-letter code in beige (observed NT ligand), blue (predicted Hc-TeTN ligand) or magenta (d5 of TrkB from the crystal complex with NT (magenta) [1HCF] [6]. Even if the main chain fragments (nearby the black C-Term label CT) of arginine (beige R) and lysine (blue K) appear to be in proximity (at least locally in this binding patch), they soon follow completely different directions since the overall folds of both proteins are totally different. To the right and topmost it can be appreciated how two asparagines (blue N+N) replace the intensive bonding network the arginine (beige R) provides. At that site both backbones (blue and beige) fairly differ because both asparagines only come into the interaction spot by their (omega) head groups, the positions of their C alpha atoms on the backbones are absolutely not coinciding with the orientation and position of the analogous arginine. The hydrophobic zone of the binding patch is colored in green. Moreover, details about the multiple segment alignment step which was carried out by *Clustal* (cluster alignment tool [21–23]) in the Bioinformatics module of Vega ZZ [1] are documented in Figure 7.

Table S1. Listing of key steps of the in-silico study procedures and work flow with prospective comments. The row-wise listing reflects the progression of the work.

Key aspects	Concise comments (see text for details and references)
Medical need of a causal cure against clostridial neurotoxins	Only temporary protection exists by prophylactic vaccination; or general life-sustaining interventions in intensive care units to treat unprotected acute intoxications with poor survival rates.
No specific antidote against CNTs exists	Clostridial neurotoxins are active in the picomolar range and recognized and internalized upon binding by cell surface components.
Dual binding mode established	But ganglioside binding cannot explain all experimental observations concerning CNT activities upon cell binding and uptake.
Triple binding mode postulated	The presence of a protein as a third binding component should resolve the observed inconsistencies in the literature.
CNTs binding to NTRs postulated	NTs bind to Trk. CNTs could bind to Trk as the missing protein component for specific ligand recognition and internalization. Crystal structures of NT-Trk complexes identified the ligand binding site (BS) on ectodomain D5 of Trk (=NTRs).
d5 binding CNTs postulated	Hypothetical CNTs binding to D5 competing with NTs as natural ligands of Trk. NTs are co-crystallized with D5 as homo or heterodimers. A monomeric biological unit of CNT-d5 was assumed because MW and Vol of two NT molecules resemble one CNT.
No homology found between CNTs and NTs	Despite the assumed common BS, dimeric NTs and monomeric CNTs show neither similarity nor any conserved region in their sequences or structures. The postulated CNT - D5 binding mechanism must occur in analogy to the observed NT binding to d5.
Analogy between CNTs and NTs postulated	Evolutionary convergence between two structurally unrelated ligands (CNTs, NTs) due to a common function. Both experience the same pressure as competitive receptor binders. Crystallographic data exist for both proteins, so convergence is amenable for modeling.

Analogy approach for PPI modeling feasible	The unknown protein-protein interaction between CNTs and Trk can be inferred by analogy from the PPI of NT-NTR crystal complexes (at the BS on D5 of Trk).
Antidote against CNTs based on PPI postulated	Once the PPI between CNTs and NTRs elucidated, a short cyclic peptide could imitate that PPI patch to become an antidote as a scavenger or suicide molecule to prevent CNTs to bind to D5 of Trk.

PPI study by MD between antidote and D5 of TrkB	Ligand - receptor docking and molecular dynamics simulations to study PPI between antidote peptide and target protein TrkB.
Synthesis and bioassays	See also patent application for more experimental details.

Table S2. Listing of the observed (2nd& 3rd columns) and calculated (3rd& 4th columns) non-bonded interactions of essential residues (One-letter code^{ID}) in the observed (first column) or calculated (last column) interfaces. Observations from crystal structures (1WWW [8], 1HCF [6]); TeTN = HC; (w, p) Hb = (water-mediated, polar) Hydrogen bond; +- = ion bridge; no = no observation; np=nonpolar, hydrophobic; bb = backbone. Asterisk * marks the three residues proposed for SDM studies. $Y^{266}A$ (Y^{1129}), $K^{311}A$ (K^{1174}) and $E^{343}A$ (E^{1206}) (residue numbers as in 1A8D [11], or in parenthesis from the total Hc domain sequence in UniProtKB/Swiss-Prot P04958-2 [14]).

Interface for columns 2&3	Ligand residues	Receptor residues	Ligand residues	Interface for columns 3&4
Observed non-bonded interaction for columns 2&3 [6,8]	Two observed complexes with ligands: NGF / NT4/5 [6,8]	NT - binding domain D5 of NTR: TrkA/B/C [6]	Computed complex with TeNT [11]	Computed non-bonded interaction for columns 3&4 (this study)
no/no	no/no	$T^{325}/S^{327}/S^{345}$	N^{341}	Hb/Hb/Hb

no/no	no/no	$S^{326}/K^{328}/K^{346}$	E^{264} E^{343} *	no/+-/+- no/+-/+-
no/npHb/weak pHb	no/no $E^{35}/E^{37}+R^{114}$	$F^{327}/Y^{329}/I^{347}$	S^{338} E^{343}	no/Hb/no no/Hb/no
+-/+-	R^{103}/R^{114}	$N^{349}/D^{349}/N^{366}$	K^{311} *	pHb/+-/pHb
Hb/Hb	H^{84}/Q^{94}	$Q^{350}/N^{350}/K^{367}$	E^{264} Y^{266} * K^{311}	no/no/+- Hb/Hb/pHb pHb/pHb/no
wHb/Hb	H2O/E^{13}	$H^{297}/H^{299}/R^{316}$* (* and not adjacent L^{315} or H^{317})	E^{447}	pHb or +-/pHb or +- /+-
np/Hb	I^{6}/R^{10} (bb)	$L^{333}/H^{335}/Y^{353}$* (* and not adjacent Y^{352}, H^{349})	D^{452}	no/pHb or +-/ pHb or +-

<u>The modeling of the tetanus protein by homology</u>

The high percentage identity (% id = 40) between tetanus and botulinum neurotoxins indicates that the generation of a 3D model of the complete TeNT from the BoNT serotype B template (PDB code: 1EPW [12]) was reliable considering the total length of approx. 1300 residues (**Figure S8**). The two

putative Trk binding domains (Hc) are identical in 31% of the 204 residue-long Hc-TeNT segment (PDB code: 1FV2_A [9]) against the 211 residues on Hc_BoNT/B (1EPW_A [12]).

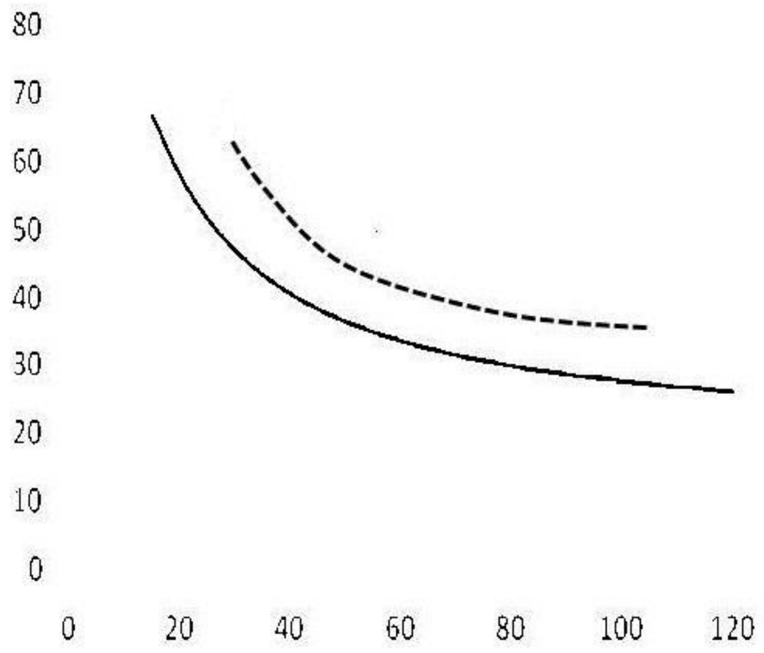

Figure S8. Plot between amino acid sequence length (x axis) against the % id between target and template aa seqs (y axis). The line marks the minimal sequence identity score (y axis) necessary for reliable generation of 3D models of the protein target for a given length (x value). Below this threshold (line) lies the twilight zone where homology models of targets become erratic. The dotted line represents the threshold for sequence - function relationships.

Assuming the common function as Trk competitors, the primary sequences of NTs and TeNT were analyzed by a MSA study. No homology was detected but this situation is not as surprising as it seems at first sight since the proteins are not even remotely related belonging to prokaryotic vs eukaryotic organisms. Their identity scores lie in an area below the threshold (**Table S3**). This area is called the twilight zone where the success rate of correct protein target structure prediction based on (poorly) homologous 3D templates become notoriously unreliable (**Figure S8**).

Table S3. Calculated sequence identity scores (in % values) for four structurally known NTs in complex with NTR. The reference molecule is the 204 residue-long Hc terminal segment of TeNT (id score = 100 %) [1FV2] [9] by Clustal W [21–23].

PDB_chain	Protein	Length	%
1B8M_A	BDNF	119	8
3BUK_A	NT3	119	7
1HCF_A	NT4	130	6
1SG1_A	beta-NGF	120	5

General aspects of protein – protein interaction

Conventional small organic ligand-receptor docking concepts cannot be applied directly without considering protein size, shape and residue composition. Adopted tools for high precision docking require the knowledge of the protein-protein site of binding due to limited computational resources for sampling conformation-dependent atomic interactions upon binary complex formation. Low precision tools, sometimes called extensive docking, are designed to explore larger surfaces to pinpoint the binding site in the first place. False positive solutions must be expected due to the trade-off made between simulation speed against algorithm complexity for search space and ranking of poses (scoring). The practical experiments called critical assessment of protein interactions (CAPRI) send a clear warning to all modelers that the more conformations changes take place the more difficult it becomes to predict PPIs in an unsupervised manner. Two mayor tenets have to be considered:

(i) However, when two bound proteins are studied in docking simulations, the studies are less error-prone, almost fool-proof and can be carried out in a straightforward manner: the two proteins of the complex appear with their structures ready for binary association (bound states). Their 3D models are extracted from known binary (crystal) complexes. When compared to existing apo forms (unliganded) or other liganded complexes of these proteins, geometrical changes can be ascribed to

induced fit events: main and side chain conformational changes, secondary structure modifications, loop arrangements or domain rotations. The bound state is amenable to rigid body docking and much resembles the validation of drug candidate screens by self-docking of a small organic molecule into its binding site (CAPRI).

(ii) When unbound proteins are studied, this means that they have not yet seen each other, and they have not adopted binding conformations. Both complex subunits appear under structures which have never "seen" or contacted each other (unbound states). Three constellations influence the fate of docking: (1) both proteins were retrieved as unbound or free structures, (2) one is, or (3) both are complexed to another binder molecule.

A wide bibliographic survey carried out by Saladin & Prevost, Melquiond & Bonvin, and London & Schueler-Furman gives rise to caution concerning the reliability of fully automated docking of PPIs [24,25].

General aspects of our analogy approach to model the unknown protein - protein interaction

To date, predicting protein - protein interactions are not flawless, and much progress has to be achieved to improve reliability and define applicability ranges. Current interface prediction tools exploit primary sequence information or protein structure data, sometimes in combination with physicochemical propensities to determine what distinguishes interface- active from inactive residues on the protein surface. The algorithms used thereby use mostly either empiric look-up tables derived from experimental PPI, or probabilistic inference techniques (Bayesian, ANN, EM, HMM, MC etc.) [26–28]. Moreover, the amino acid abundance (and their nonbonded interactions to target) engaged in protein-protein interactions was found to be very different to the interaction between drug-like ligands and protein targets: Asp, Arg, Ile, Pro, Trp or Tyr vs. His, Met, Phe, Trp, or Tyr [26,29,30].

Like secondary structure prediction tools were trained to recognize segments with 13 residues (machine learning) because shorter or larger search windows may introduce over- proportionally more noise – now the sequence-based PPI prediction tools also face that kind of problems [27]. The drawback of predictions tools based on 3D structural data lies in side chain flexibilities and the active

conformation on occasions is difficult to foresee, like rotations of loop segments, local main chains or even entire domains [26,31]. It was assumed that the N- to C-term axis of D5 is oriented top-down just above the cell membrane since the ligand binding domain D5 is the closest of all five Trk ectodomains to the membrane surface [6,19,29,32,33, 34].

After the inspection of the protein surfaces between neurotrophin ligand and its receptor TrkB the analogy to the neurotoxin as potential ligand was modeled (**Figures S9** and **S10**).

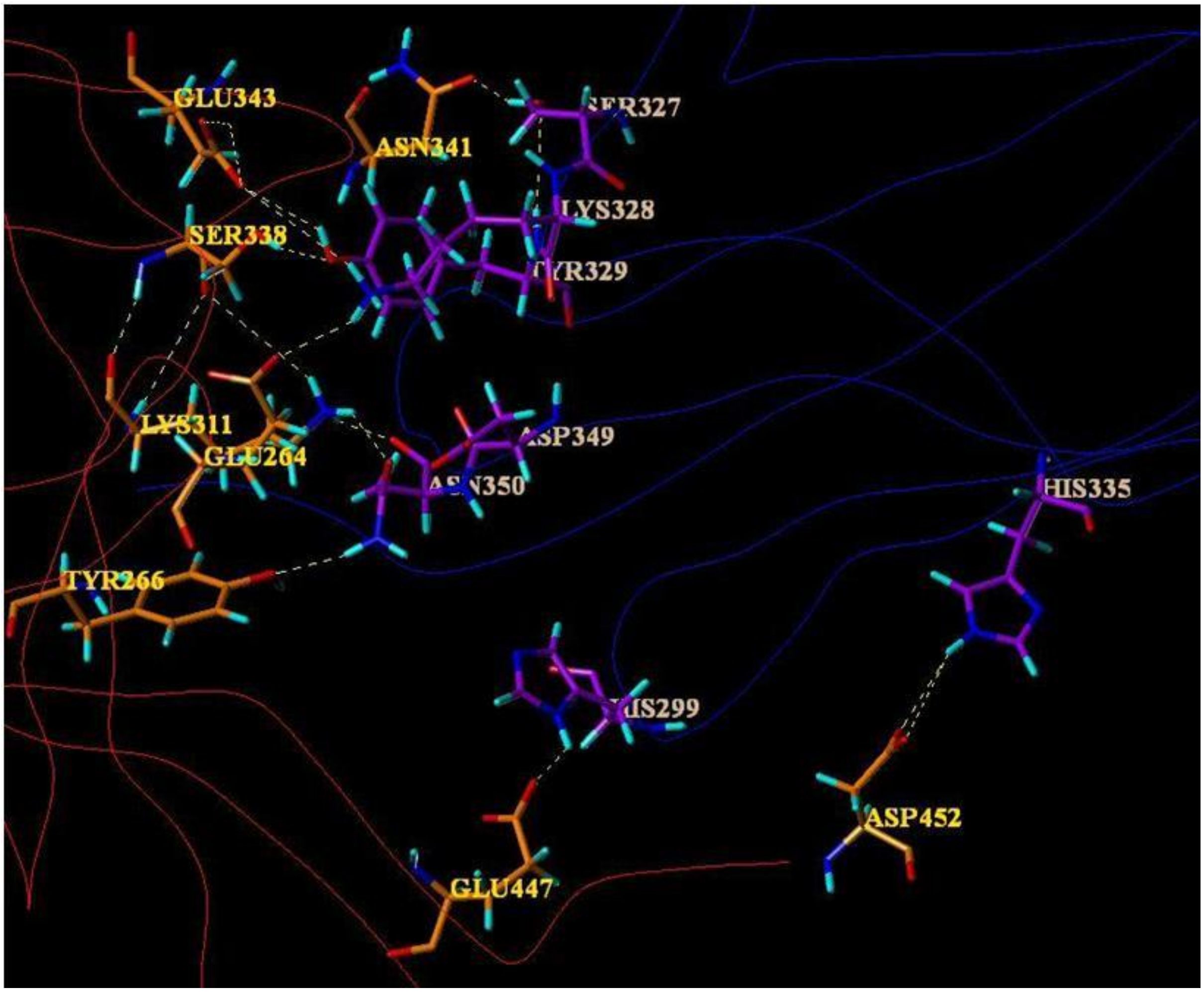

Figure S9. The three-dimensional model displays the amino acids on both sides of the ligand - receptor interface between the proposed analogous interface between Hcc-TeNT and D5 of TrkB

(right-hand side). The hydrogen-bonding network is displayed (dashed yellow lines). Color code: Carbon atoms of TeNT in orange, O atoms in red, N atoms in blue, polar H atoms in light blue. All C atoms of binding domain D5 in purple color. Backbone of D5 in dark-blue lines. The backbone line of ligand TeNT (to the left) is in orange color. The strong non- bonded interaction through hydrogen bonds and ion bridges becomes evident (dashed lines). The id labels follow the positional numbers (**Figure S8** and **Table S2**).

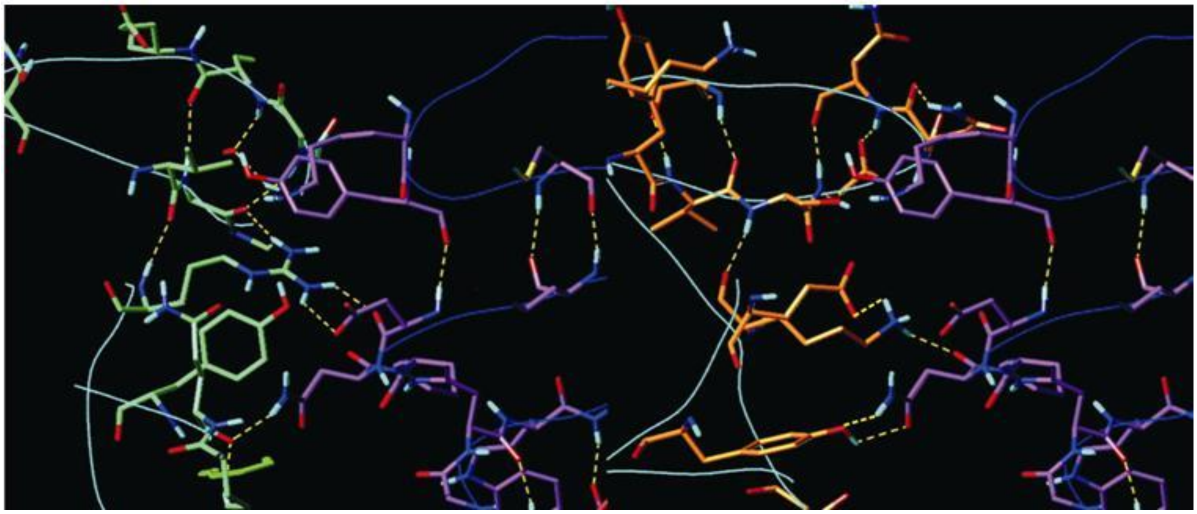

Figure S10. Two 3D models depict the analogy between observed and computed binding (for details, refer to **Table 2**). Observed neurotrophin (NT) binding to TrkB (left-hand half) and the analogous interface between Hc-TeNT and D5 of TrkB (right-hand half). The hydrogen- bonding network is displayed (dashed yellow lines). Color code: Carbon atoms of NT in green, C atoms of TeNT in orange, O atoms in red, N atoms in blue, polar H atoms in light blue. All C atoms of binding domain D5 in purple color, D5 backbone in dark blue lines. The backbones of both ligands (NT to the left and TeNT to the right) are light-blue lines. The analogy of both binding modes becomes evident. The ligands folds stretch out like a "protruding flap", seen in the upper-left part of each panel. Both ligands show backbones forming the interface to D5 (vertical light blue lines).

At the modeling stage of our study, the designed cyclic peptide was compared to shorter and longer versions. It was found that the originally 14 residue long half was best suited as a starting point for synthesis and experimental tests (lead compound). Longer peptide chains would only be necessary

to account for long range effects like in protein folding processes. Shorter peptides could make the ring too stiff and therein exclude the productive conformations. Many functions are maintained by 5 to 15 residues forming a local string, for instance at enzymatic sites, ligand recognition motifs or secondary structures. No wonder, a typical evaluation window of bioinformatics tools for local pattern prediction has a width of 13 amino acids to screen through the sequences (**Figures S11 and S12**).

```
  __disulfide bridge___
 |                     |
GCLQLDNPTHMNNGMLQLDNPTHCNNG
123456789012345678901234567
1        10        20     27
```

Figure S11. Schematic drawing of the studied cyclic D5 peptide molecule (proposed antidote). The amino acid sequence is given in one-letter code. A disulfide bond is formed between two cysteines amino acids, Cys2 and Cys24. The N-terminal amino acid Gly1 is exocyclic. At the C-terminal part Asn25, Asn26 and residue Gly27 are outside the ring. Of note, the last two lines together give the residue id numbers. The underlined four letters in Italics (DN, MN) are the four D5 receptor residues forming the PPI with the ligands (Asp349 - Asn350 and Met354 - Asn355).

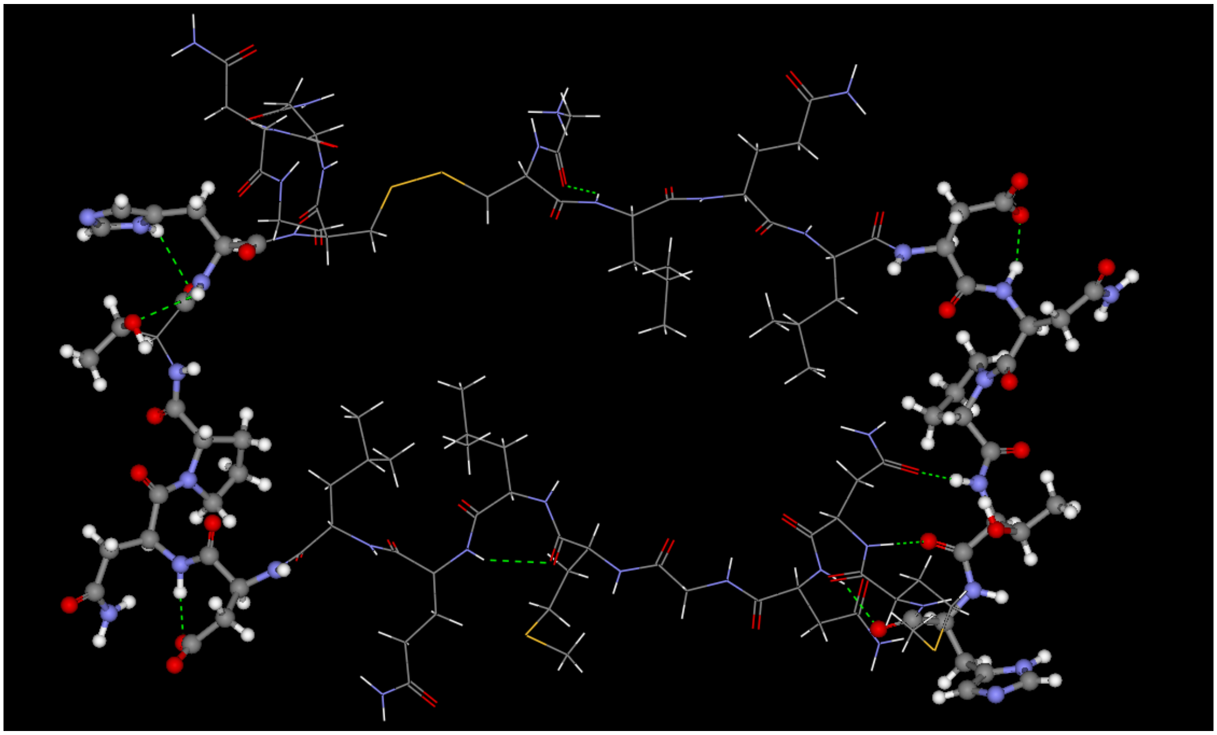

Figure S12. 3D model of cyclic D5 peptide. The 27 residue long linear oligopeptide is ring-closed through a disulfide bridge (yellow SS-bond, upper mid-section). Hydrophobic side chains are oriented toward the ring center. Two monoanionic carboxylic end groups are visible (upper right and lower left corners).

Duplicating a binding segment as well as introducing a Cys-Cys bond for ring formation by means of a specific point mutation has converted the natural binding segment of D5 into our starting point for experimental studies for the proposed antidote activity (**Figures S11** and **S12**). Of note, for more details, refer to patent application "Peptide for use in the treatment of disease caused by clostridium neurotoxins", WO 2017050816 A1).

The risk of allergic reactions of the treated organism appears to be fairly reduced because the amino acid composition of the natural peptide is kept whereas only two residues switch their positions (**Figure S13**).

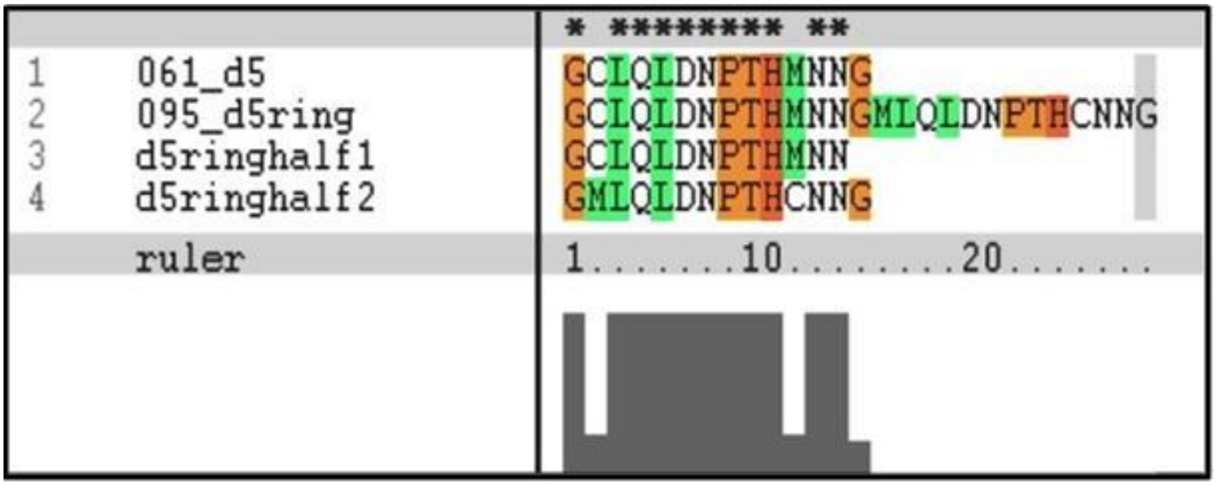

Figure S13. Alignment study of the amino acid sequences. The discovered neurotoxin-interacting part of the human receptor domain D5 of TrkB is aligned with the D5 peptide (proposed antidote construct). The identical parts and the mutated amino acids can be appreciated in four lines.

line 1, original segment from D5 of TrkB, around Asn350, 14 residues long
line 2, invented linear peptide ring = antidote, 27 residues long
line 3, first half of line 2, to compare it to Line 1
line 4, second half of line 2 to compare it to Lines 1 and 3
bottom, the grey bars show the almost total identity and the Met/Cys mutation.

ABBREVIATIONS

1AF9, TeNT target ligand;
1A8D, TeNT target ligand;
1HCF, D5 of NT4/5 ligand and D5 of NTR TrkB;
AKA, also known as;
BDNF, brain-derived neurotrophic factor;
BoNT/A, botulinum neurotoxin, A type;
BoNT/B, botulinum neurotoxin, B type;
CNT, clostridial neurotoxin: (e.g. TeNT and BoNT);
C-fragment, PDB code: 1A8D, chain of tetanus neurotoxin, cf. HC and HCR/T;
D5, The fifth extracellular domain of the human nerve cell membrane receptor TrkB;
D5 peptide, synthetic cyclic peptide extracted from D5 as the target, cf. Figure S11;
H4 peptide, synthetic peptide with amino acids of D5 peptide in random sequence;
Hc, heavy chain of tetanus neurotoxin, AKA "fragment-C";
Hcc, carboxyl-terminal domain of the heavy chain (AKA "HC");
Hc-Mut, the triple mutant with mutated residues Y266A K311A E343A;

Hcc-TeNT, carboxyl-terminal domain of the heavy chain of tetanus neurotoxin;
HCR/T, carboxyl-terminal receptor-binding domain of tetanus neurotoxin, cf. Hcc;
IC_{50}, half maximal inhibition concentration;
MSA, Multiple sequence alignment (studies);
NGF, nerve growth factor, cf. NT;
NT, Neurotrophins: BDNF, NT-3, NT-4/5, and NGF;
NTR, neurotrophic cell surface receptors ($p75^{NTR}$, TrkA, B and C); $p75^{NTR}$, low-affinity NTR which binds all NTs;
TeNT or TeNT, tetanus neurotoxin (Synonyms: tetanus toxin, tetanospasmin, spasmogenic toxin);
Trk, tropomyosin receptor kinase or tyrosine kinases of class VII of the “receptor tyrosine kinases” superfamily (RTK: TrkA, B, and C); TrkAIg(2), second Ig-like domain binding NGF = D5 of TrkB; TrkB, TRK-B = TrkB receptor = TrkB tyrosine kinase = BDNF/NT-3 growth factors receptor = neurotrophic tyrosine kinase receptor type 2.

REFERENCES OF SM

1. Pedretti, A.; Villa, L.; Vistoli, G. VEGA – An Open Platform to Develop Chemo-Bio-Informatics Applications, Using Plug-in Architecture and Script Programming. *J Comput Aided Mol Des* **2004**, *18*, 167–173, doi:10.1023/B:JCAM.0000035186.90683.f2.
2. Ausiello, G.; Cesareni, G.; Helmer-Cittarich, M. ESCHER: A New Docking Procedure Applied to the Reconstruction of Protein Tertiary Structure. *PROTEINS: Structure, Function, and Genetics* **1997**, *28*, 556–567.
3. Pettersen, E.F.; Goddard, T.D.; Huang, C.C.; Couch, G.S.; Greenblatt, D.M.; Meng, E.C.; Ferrin, T.E. UCSF Chimera?A Visualization System for Exploratory Research and Analysis. *J. Comput. Chem.* **2004**, *25*, 1605–1612, doi:10.1002/jcc.20084.
4. Krivov, G.G.; Shapovalov, M.V.; Dunbrack, R.L. Improved Prediction of Protein Side-Chain Conformations with SCWRL4: Side-Chain Prediction with SCWRL4. *Proteins* **2009**, *77*, 778–795, doi:10.1002/prot.22488.
5. Berman, H.M. The Protein Data Bank. *Nucleic Acids Research* **2000**, *28*, 235–242, doi:10.1093/nar/28.1.235.
6. Banfield, M.J.; Naylor, R.L.; Robertson, A.G.S.; Allen, S.J.; Dawbarn, D.; Brady, R.L. Specificity in Trk Receptor:Neurotrophin Interactions. *Structure* **2001**, *9*, 1191–1199, doi:10.1016/S0969-2126(01)00681-5.

7. Ultsch, M.H.; Wiesmann, C.; Simmons, L.C.; Henrich, J.; Yang, M.; Reilly, D.; Bass, S.H.; de Vos, A.M. Crystal Structures of the Neurotrophin-Binding Domain of TrkA, TrkB and TrkC 1 1Edited by I. A. Wilson. *Journal of Molecular Biology* **1999**, *290*, 149–159, doi:10.1006/jmbi.1999.2816.
8. Wiesmann, C.; Ultsch, M.H.; Bass, S.H.; de Vos, A.M. Crystal Structure of Nerve Growth Factor in Complex with the Ligand-Binding Domain of the TrkA Receptor. *Nature* **1999**, *401*, 184–188, doi:10.1038/43705.
9. Fotinou, C.; Emsley, P.; Black, I.; Ando, H.; Ishida, H.; Kiso, M.; Sinha, K.A.; Fairweather, N.F.; Isaacs, N.W. The Crystal Structure of Tetanus Toxin Hc Fragment Complexed with a Synthetic GT1b Analogue Suggests Cross-Linking between Ganglioside Receptors and the Toxin. *Journal of Biological Chemistry* **2001**, *276*, 32274–32281, doi:10.1074/jbc.M103285200.
10. Umland, T.C.; Wingert, L.M.; Swaminathan, S.; Furey, W.F.; Schmidt, J.J.; Sax, M. Structure of the Receptor Binding Fragment HC of Tetanus Neurotoxin. *Nat Struct Mol Biol* **1997**, *4*, 788–792, doi:10.1038/nsb1097-788.
11. Knapp, M.; Segelke, B.; Rupp, B. The 1.61 Angstrom Structure of the Tetanus Toxin Ganglioside Binding Region: Solved by MAD and Mir Phase Combination. *Am. Cryst. Assoc. Abstr. Papers (Annual Meeting)* **1998**, *25*, doi:10.2210/pdb1A8D/pdb.
12. Swaminathan, S.; Eswaramoorthy, S. Structural Analysis of the Catalytic and Binding Sites of Clostridium Botulinum Neurotoxin B. *Nat Struct Biol* **2000**, *7*, 693–699, doi:10.1038/78005.
13. Jayaraman, S.; Eswaramoorthy, S.; Kumaran, D.; Swaminathan, S. Common Binding Site for Disialyllactose and Tri-Peptide in C-Fragment of Tetanus Neurotoxin. *Proteins* **2005**, *61*, 288–295, doi:10.1002/prot.20595.
14. The UniProt Consortium UniProt: A Worldwide Hub of Protein Knowledge. *Nucleic Acids Research* **2019**, *47*, D506–D515, doi:10.1093/nar/gky1049.
15. Pundir, S.; Martin, M.J.; O'Donovan, C.; UniProt Consortium UniProt Tools. *Curr Protoc Bioinformatics* **2016**, *53*, 1.29.1-1.29.15, doi:10.1002/0471250953.bi0129s53.
16. Baxevanis, A.D. *Current Protocols in Bioinformatics*; John Wiley and Sons: New York, 2004;
17. *Fmoc Solid Phase Peptide Synthesis: A Practical Approach*; Chan, W.C., White, P.D., Eds.; The practical approach series; Oxford University Press: New York, 2000; ISBN 978-0-19-963725-6.
18. Cubí, R.; Candalija, A.; Ortega, A.; Gil, C.; Aguilera, J. Tetanus Toxin Hc Fragment Induces the Formation of Ceramide Platforms and Protects Neuronal Cells against Oxidative Stress. *PLoS ONE* **2013**, *8*, e68055, doi:10.1371/journal.pone.0068055.
19. Pattarawarapan, M.; Burgess, K. Molecular Basis of Neurotrophin−Receptor Interactions. *J. Med. Chem.* **2003**, *46*, 5277–5291, doi:10.1021/jm030221q.
20. REF OMITTED
21. Thompson, J.D.; Higgins, D.G.; Gibson, T.J. CLUSTAL W: Improving the Sensitivity of Progressive Multiple Sequence Alignment through Sequence Weighting, Position-Specific Gap Penalties and Weight Matrix Choice. *Nucleic Acids Res.* **1994**, *22*, 4673–4680, doi:10.1093/nar/22.22.4673.

22. Sievers, F.; Wilm, A.; Dineen, D.; Gibson, T.J.; Karplus, K.; Li, W.; Lopez, R.; McWilliam, H.; Remmert, M.; Söding, J.; et al. Fast, Scalable Generation of High-quality Protein Multiple Sequence Alignments Using Clustal Omega. *Mol Syst Biol* **2011**, *7*, 539, doi:10.1038/msb.2011.75.
23. Larkin, M.A.; Blackshields, G.; Brown, N.P.; Chenna, R.; McGettigan, P.A.; McWilliam, H.; Valentin, F.; Wallace, I.M.; Wilm, A.; Lopez, R.; et al. Clustal W and Clustal X Version 2.0. *Bioinformatics* **2007**, *23*, 2947–2948, doi:10.1093/bioinformatics/btm404.
24. *Protein-Protein Interactions in Drug Discovery*; Dömling, A., Ed.; Methods and principles in medicinal chemistry; Wiley-VCH: Weinheim, 2013; ISBN 978-3-527-33107-9.
25. *Protein-Protein Complexes: Analysis, Modeling and Drug Design*; Zacharias, M., Ed.; Imperial College Press ; Distributed by World Scientific Pub: London : Hackensack, NJ, 2010; ISBN 978-1-84816-338-6.
26. Najmanovich, R.; Kuttner, J.; Sobolev, V.; Edelman, M. Side-Chain Flexibility in Proteins upon Ligand Binding. *Proteins* **2000**, *39*, 261–268, doi:10.1002/(sici)1097-0134(20000515)39:3<261::aid-prot90>3.0.co;2-4.
27. Zhou, H.-X.; Qin, S. Interaction-Site Prediction for Protein Complexes: A Critical Assessment. *Bioinformatics* **2007**, *23*, 2203–2209, doi:10.1093/bioinformatics/btm323.
28. Peng, X.; Wang, J.; Peng, W.; Wu, F.-X.; Pan, Y. Protein-Protein Interactions: Detection, Reliability Assessment and Applications. *Brief Bioinform* **2017**, *18*, 798–819, doi:10.1093/bib/bbw066.
29. Sillerud, L.; Larson, R. Design and Structure of Peptide and Peptidomimetic Antagonists of Protein-Protein Interaction. *CPPS* **2005**, *6*, 151–169, doi:10.2174/1389203053545462.
30. Gallina, A.M.; Bork, P.; Bordo, D. Structural Analysis of Protein-Ligand Interactions: The Binding of Endogenous Compounds and of Synthetic Drugs: STRUCTURAL ANALYSIS OF PROTEIN-LIGAND INTERACTIONS. *J. Mol. Recognit.* **2014**, *27*, 65–72, doi:10.1002/jmr.2332.
31. Lensink, M.F.; Méndez, R. Recognition-Induced Conformational Changes in Protein-Protein Docking. *Curr Pharm Biotechnol* **2008**, *9*, 77–86, doi:10.2174/138920108783955173.
32. Windisch, J.M.; Marksteiner, R.; Lang, M.E.; Auer, B.; Schneider, R. Brain-Derived Neurotrophic Factor, Neurotrophin-3, and Neurotrophin-4 Bind to a Single Leucine-Rich Motif of TrkB. *Biochemistry* **1995**, *34*, 11256–11263, doi:10.1021/bi00035a035.
33. Keskin, O.; Tuncbag, N.; Gursoy, A. Characterization and Prediction of Protein Interfaces to Infer Protein-Protein Interaction Networks. *Curr Pharm Biotechnol* **2008**, *9*, 67–76, doi:10.2174/138920108783955191.
34 Janin, J. Chapter 1: X-ray Study of Protein – Protein Complexes and Analysis of Interfaces. Domling, A. Protein-protein interactions in drug discovery. (Editor) Wiley-vch, 2008, Bookchapter 1 56. ISBN, 9783527331079.
